# Factors Determining the Choice of a Career in Geriatrics among Students in Geriatric in-Hospital Training: A Prospective Study of 74 Medical Students

**DOI:** 10.3390/geriatrics5040087

**Published:** 2020-11-04

**Authors:** Valentine Nuss, Jérémy Barben, Caroline Laborde, Jérémie Vovelle, Martha Deidda, Anca-Maria Mihai, Alain Putot, Patrick Manckoundia

**Affiliations:** 1 “Pôle Personnes Âgées”, Hospital of Champmaillot, Dijon Bourgogne University Hospital, 21079 Dijon, France; valentine.nuss@chu-dijon.fr (V.N.); jeremy.barben@chu-dijon.fr (J.B.); carolinelaborde88@gmail.com (C.L.); jeremie.vovelle@chu-dijon.fr (J.V.); martadeidda@yahoo.it (M.D.); anca-maria.mihai@chu-dijon.fr (A.-M.M.); alain.putot@chu-dijon.fr (A.P.); 2 INSERM U-1093, Cognition, Action and Sensorimotor Plasticity, University of Burgundy Franche-Comté, 21000 Dijon, France

**Keywords:** geriatrics, clinical in-hospital training, medical education, medical students

## Abstract

To understand why students in the 2nd cycle of medical studies choose to complete a Diploma of Specialized Studies (DSS) in geriatrics, we conducted a study to identify the factors influencing the choice of a future specialty. In addition, we assessed the impact of clinical in-hospital training (CIHT) in a geriatric hospital on the students’ selection of their future specialty. We included all students who completed CIHT in the geriatric facility of our University Hospital between 1 May and 31 October 2018. Data were collected using a two-part questionnaire: one part was given before CIHT and the other after. The students were classified into two groups: those considering a career in geriatrics (CIG) before CIHT, forming the group DSS geriatrics+ (GDSSG+), and those not considering it, constituting the group DSS geriatrics− (GDSSG−). Seventy-four students aged 22 years old were included. Of these students, 26% were considering a CIG before CIHT. This rate increased significantly to 42% after CIHT (*p* = 0.04). However, none of the students who indicated that they were potentially interested in pursuing geriatrics before CIHT preselected geriatrics as their first option. For more than 92% of the students, the comprehensive care of geriatric patients was an asset. The main drawbacks were diagnostic and therapeutic limitations (60% of students), then managing aging, disability, and neurocognitive disorders (55% of students). After CIHT, the view of geriatrics improved by 74%. In conclusion, geriatric CIHT improves students’ opinions of geriatrics and increases the number of students considering a CIG. However, geriatrics still suffers from a lack of prestige.

## 1. Introduction

Life expectancy is growing worldwide, resulting in a growing number of frail elderly individuals [1].

Care of the elderly requires an adequate number of specialists in geriatrics, but in France, the number of specialists in this domain, an estimated 1997 physicians, remains low [2]. In 2018, the mean age of geriatricians in France was 50.3 years [2]. The density of this specialty was 3 geriatricians per 100,000 adults aged ≥75 years, with in some regions, less than 1 geriatrician per 100,000 adults ≥75 years. That would provide a shortage of geriatricians in France [2]. More than ¾ of geriatricians were exclusively employed in hospitals, 17% were exclusively employed in nonhospital activities (medical coordination in nursing homes, public administrations, private institutions, etc.), 3% had an exclusive private practice, and 3% had a mixed activity (an activity as an employed and a private practice) [2].

As explained by Prud’homm and colleagues [3], French medical studies are divided into three cycles. The 1st cycle lasts 3 years and the 2nd lasts 4 years. We are interested in the latter in this study. The 2nd cycle awards a qualification corresponding to a Master’s degree in medicine (MM), is divided into MM1 for year 4, MM2 for year 5, and MM3 for year 6. During these 3 years, theoretical and practical training are combined to include clinical in-hospital training (CIHT) usually lasting 6 to 8 weeks and undertaken in various hospital departments as well as in the private medical practice of a general practitioner. This cycle ends with the French national ranking exam, which determines the specialty and the city of practice as an intern. The last phase of medical studies, the 3rd cycle, spans a period of 3–6 years, ending with a Diploma of Specialized Studies (DSS). In France, geriatrics became a DSS in 2017.

Despite the obvious need for qualified personnel, hospitals struggle to recruit geriatricians and particularly to attract young physicians to this specialty [4]. It appears that medical students (MS) have little interest in pursuing a career in geriatrics (CIG).

It seems important to understand why students tend to dismiss a CIG. Our hypotheses include a lack of information and/or knowledge of this specialty, the fear of a specialty judged as being relational and insufficiently technical, and the fear of having to deal with difficult end-of-life situations and death on a regular basis.

We conducted this study to identify the reasons for which MS chose a DSS in Geriatrics (DSSG) or not, and the factors influencing their choice of specialty. We also evaluated the impact of CIHT in geriatrics on the choice of specialty.

## 2. Methods

We conducted a prospective, descriptive, single-center study of MS in the geriatric center of a university hospital.

This study was outside the field of Jardé’s law and Ethics Committee approval was considered unnecessary under French law.

Oral consent was obtained from all participants, and the data were processed anonymously.

We included all MS who completed a CIHT in our geriatric facility in the 2nd cycle of their studies between 1 May 2018 and 31 October 2018. Our facility includes an ambulatory geriatric department, acute geriatric units, geriatric rehabilitation units, and a nursing home.

There was no exclusion criterion.

The participants were divided into two groups: those who were considering a CIG before CIHT, group DSSG+ (GDSSG+), and those who were not considering it, group DSSG− (GDSSG−). We also analyzed student views regarding the advantages/disadvantages of CIG at two time points (before and after CIHT) in both groups. Finally, we assessed the number of MS in the two groups before and after CIHT.

An anonymous two-part questionnaire was given to MS at the beginning and the end of CIHT. The questionnaire was developed by three members of our team—among them, a statistician. After selecting the questions that seemed most relevant to us, the questionnaire was tested by administering it to a sample of students before the start of the study. The questionnaire was administered in paper format. For each student, the following were collected:

− Demographic data: age (years), gender, year of study.
− Preferred specialty after the French national ranking exam: medical practice type and conditions intended, considered medical specialties, and factors influencing the choice of specialty.
− Interest in geriatrics before CIHT: Was it the first experience in a geriatric environment? Was the choice of CIHT in a geriatric center related to an interest in geriatrics and knowledge of the DSSG?
− Interest in geriatrics before and after CIHT: advantages/disadvantages of geriatrics, and CIG considered or not.
− Interest in geriatrics after CIHT: changes in view of geriatrics (geriatric training, bedside teaching, immersion in the care team, contact with patients); improvement or not in medical management of the elderly; quality of supervision; quality of care team’s supervision; sufficient immersion in geriatric culture; would or would not recommend CIHT in geriatrics; and ways of improving CIHT.

The survey had no impact on the outcome of CIHT. Students were assured that their responses would not affect the CIHT validation. The questionnaires were analyzed at the end of inclusions.

We described the data collected on the criteria influencing students’ choice of a future specialty before CIHT, the strengths and weaknesses of geriatrics before and after CIHT, and the change in student views of geriatrics after CIHT in the total study population and the two groups. Finally, we assessed whether CIHT had an effect on whether MS were considering a CIG.

Chi-square and Fisher tests were used to analyze categorical variables. Student’s *t*-test was used for quantitative variables. Statistical significance was set at *p* < 0.05.

## 3. Results

### 3.1. Total Population

#### 3.1.1. Before CIHT

##### Participants’ Characteristics

Seventy-four participants (43 women) aged 22 ± 1.69 years were included.

Forty-two percent of the MS were in MM1, 39% in MM2, and 19% in MM3. Furthermore, 44% of the MS completed their CIHT in acute geriatric units, 34% in the geriatric rehabilitation units, and 22% in the nursing home. Of the MS, 9% were considering in-hospital medical practice, 34% were attracted to private practice, 30% to a mixed medical practice, and 27% were undecided. The geriatric CIHT was a choice by conviction for 70%. For 42%, it was the first experience in geriatrics.

Fifty-seven students (77%) had already chosen their future specialty: 27 had a preference for general medicine, 16 preferred another medical specialty, 6 selected surgery, 6 selected gynecology or pediatrics, and 2 selected emergency medicine. None of these MS listed geriatrics as a first choice.

Forty-five students (61%) were aware of the DSSG, while 71 (96%) felt that the information received was insufficient or nonexistent, expressing a desire for more information (Table 1).

##### Criteria Influencing Choice of Future Specialty

The three most relevant criteria for the choice of specialty were the varied activity in the specialty for 89%, intellectual challenge for 73%, and the possibility of working in a private practice for 64% (Table 2).

#### 3.1.2. Pre- and Post-CIHT Questionnaire Comparison

Comprehensive patient management, the interest in ethical issues, the multidisciplinary teamwork, and the varied activity were the strengths of geriatrics. There were no significant differences between pre- and post-CIHT (Table 3).

The frustration of having to limit diagnostic investigations and therapeutic options; dealing with aging, disability, and major neurocognitive disorders; contending with death; and the inability to have a private practice were the disadvantages for geriatrics. There were no significant differences between pre- and post-CIHT (Table 4).

#### 3.1.3. At the End of CIHT

Fifty-five students (74%) expressed a more positive opinion of geriatrics at the end of CIHT, and 70 agreed that it had resulted in a positive change in their approach to managing the elderly. The impact of the teaching during CIHT was found to be positive for the theoretical aspects for 60/74 MS and for the practical aspects for 59 MS. The practice of elderly care was positively impacted by theoretical content of the lessons for 63 MS, while it was positively impacted by the practical training courses for 67 MS (Table 5).

### 3.2. GDSSG+ vs. GDSSG−

#### 3.2.1. Before CIHT

At inclusion, the GDSSG+ comprised 19 MS, aged 23 ± 1.48, and the GDSSG− had 55 MS, aged 20 ± 1.77, with no difference for age (*p* = 0.86).

Significantly more MS in the GDSSG+ had a preference for in-hospital medical practice (26% vs. 4% in the GDSSG−; *p* = 0.01). More MS had a preference for private medical practice in the GDSSG− (36%) than in the GDSSG+ (26%), with no difference (*p* = 0.77). Similarly, more MS had a preference for mixed medial practice in the GDSSG+ (37%) than in the GDSSG− (27%), with no difference (*p* = 0.43).

Overall, none of the MS had chosen geriatrics as the first option for a future specialty. General medicine was the most popular first option 58% in the GDSSG+ vs. 29% in the GDSSG−, with a significant difference (*p* = 0.02). Conversely, the choice of a medical specialty as the first option was significantly higher in the GDSSG− (27%) than in the GDSSG+ (5%) (*p* = 0.05).

Significantly more MS in the GDSSG+ had chosen geriatric CIHT by interest in geriatrics (89% vs. 63% in the GDSSG−; *p* = 0.04).

#### 3.2.2. Choice of a Future Specialty

Among the criteria influencing students’ choice of their future specialty before CIHT, only the desire for high earnings was significantly greater in the GDSSG− (22%) than in the GDSSG+ (0%) (*p* = 0.03).

#### 3.2.3. Advantages/Disadvantages of Geriatrics before and after CIHT

There were no differences between the GDSSG+ and the GDSSG− concerning the advantages of geriatrics before and after CIHT (Table 3).

Poor knowledge of geriatrics was considered a disadvantage significantly more often in the GDSSG− than in the GDSSG+, both before CIHT (40% vs. 11%, respectively; *p* = 0.02) and after (38% vs. 11%, respectively; *p* = 0.03).

The social dimension of care was the only other negative point for which the difference was significant between the two groups after CIHT (25% vs. 0%, respectively; *p* = 0.02) (Table 4).

#### 3.2.4. Impact of CIHT on Students’ View of Geriatrics

Eighty-nine percent of MS in the GDSSG+ and 69% in GDSSG− improved their view of geriatrics after CIHT, with no difference between the groups (*p* = 0.13).

All (100%) MS in the GDSSG+ and 93% in the GDSSG− agreed that CIHT had improved their management of elderly patients, with no difference (*p* = 0.57).

After CIHT, 89% of MS in the GDSSG+ recommended the geriatric CIHT compared with 95% in the GDSSG−, with no difference (*p* = 0.60).

#### 3.2.5. Impact of CIHT on Whether or Not Students Would Consider a CIG

There were significantly more MS considering a CIG after CIHT (42%) than before (26%) (*p* = 0.04).

## 4. Discussion

This original study evaluates the factors influencing the potential choice of geriatrics—which has only been possible since 2017—by MS in the 2nd cycle. A similar study was recently conducted in France on postgraduate MS (3rd cycle) [3]. Our study is complementary in that it focuses on MS in the previous cycle who have not yet chosen their specialty. Few MS choose geriatrics as a specialty, even those who have completed an internship in geriatrics. It is therefore essential to find ways to promote geriatrics among MS. It has been shown that short-term mortality is reduced and physical and cognitive capacities are improved in elderly patients managed by geriatricians [5].

Although 74% of MS had overall positive change in their view of geriatrics after their CIHT, only 31% were considering specializing in geriatrics. A British study reported a similar result. While 76% considered that geriatrics had a positive impact on the lives of the elderly, only a third of MS were considering working in geriatrics [6]. Still, none of the MS in our study selected geriatrics as their first choice. Another British study reported that 4% of students decided to specialize in geriatrics [7]. In our study, 77% had already decided on their future specialty before CIHT, which is consistent with the results of an Irish study (>two thirds of students) [8]. The lack of interest in geriatrics may partly be explained by the fact that this specialty is relatively new in some countries, even nonexistent in others. A 2015 review in 31 European countries showed that geriatrics was only recognized as a distinct specialty in 61.3% of the countries [9]. In France, the DSSG started in 2017, and there are about 200 intern positions in geriatrics available through the French national ranking system. One potential reason for the lack of attractiveness of geriatrics could be the lack of exposure during the MM program. Others studies in MS have also linked growing interest for geriatrics to the increase in the number of geriatric training courses [10,11].

One of the reported downsides of geriatrics was the frustration of being limited in diagnostic investigations and therapeutic options. This result is similar to a report by Bagri et al., who described discouragement in students who did not observe the immediate effects of certain treatments [12]. Having to deal with aging, disability, and major neurocognitive disorders was another major negative point, as was dealing with death. These factors were also reported with the statement that the chronic nature of disease in the elderly made the physician’s role less attractive [12].

Not being able to practice in private medicine, also reported in previous studies [13], was another limiting factor for the choice of geriatrics and downside for half of the MS in our study. Low income was also listed as a major drawback in other studies [12,14].

The lack of prestige was a deterrent in 16–20% of the cases in our study. Meiboom et al. also found lack of prestige to be a minor deterrent [13]. Another study suggested that the lack of peer recognition had a role in maintaining this perceived lack of prestige [15]. The fact that geriatrics is a recent specialty may contribute to a general lack of familiarity, which could partly explain the lack of consideration. Nevertheless, most geriatricians believe their specialty to be indispensable and express satisfaction in their choice of career [16].

In our study, 80% of MS confirmed that that availability of geriatrics classes improved the attractiveness of this specialty. A previous study reported a similar increase in students who attended clinical learning experiences lasting more than 8 days [13]. Two other studies showed greater interest in the specialty after training programs lasting 5 weeks or 1 month [17,18]. Ahmed et al. observed that knowledge and behavior toward patients significantly improved in the 4th year students who took part in a new university CIHT program in geriatric medicine and palliative care [19].

The number of MS considering a CIG was significantly higher after CIHT, although we were unable to determine specific reasons for the increase. In the current study, MS considering a CIG listed the variety and cross-functionality of geriatrics (47%), its intellectual appeal (74%), and comprehensive management of patients (79%) as the main reasons for this choice. These results are similar to those reported in an Irish study [8] and could be explained by the values of humanity and empathy that tend to be more necessary in geriatrics than in other more technical specialties. Indeed, creating empathy among MS is associated with positive changes in attitudes toward the elderly [20]. A British study reported that students from a university attached to a health center for the elderly showed better behavior toward these individuals than other students did, even before the start of geriatric teaching [18]. For Cankurtaran et al., the attitude of MS toward the elderly is closely related to the experiences and knowledge acquired in medical school [13]. However, adding geriatrics classes to the Master’s curriculum is not always sufficient, especially since the attitudes and knowledge of students are closely related to the amount of training they receive [21].

One survey showed that among physicians who finally settled into a career as a geriatrician, only one-third had made this choice 3 years after obtaining their MM [22]. Moreover, some studies highlight that geriatricians tend to choose their specialty relatively late in their studies [7].

The main limitation of this study is its monocentric character with relatively few participants. Another limitation is the fact that only a few parameters have been taken into consideration.

Finally, some suggestions arise from our study. The CIHT in geriatrics departments could be made mandatory during the MM. Thus, all the MS in the MM would have the opportunity to experience the practice of this specialty in immersion. Another suggestion is to increase the weight of teaching of geriatrics in the MM. We also suggest that the French authorities intensify the promotion of research in geriatrics by offering more research programs specific to geriatrics. Our latest proposal is to further enhance the medical aspect of geriatrics, in order to correct the false image of a mainly medico-social specialty. After the implementation of these measures, it would be interesting to conduct a study to find out whether they had a positive impact on the potential choice of geriatrics as a future specialty by the MS in the MM.

## 5. Conclusions

Several factors influenced MS in the choice of their future specialty, with the most important being intellectual attractiveness, varied activity, and comprehensive patient care. Geriatrics still suffers from a negative image and lack of prestige.

## Figures and Tables

**Table 1:** Student characteristics at the beginning of in-hospital training.

Parameter			Total N = 74	GDSSG+N = 19	GDSSG−N = 55	*p*
Mean age (years) ± standard deviation			22 ± 1.69	23 ± 1.48	20 ± 1.77	0.86
			N (%)	N (%)	N (%)	
Female gender			43 (58)	13 (68)	30 (54)	0.29
Education level		MM1	31 (42)	10 (53)	21 (38)	0.27
		MM2	29 (39)	6 (32)	23 (41)	0.43
		MM3	14 (19)	3 (16)	11 (20)	1
Place of CIHT		Acute geriatrics unit	33 (45)	13 (68)	20 (36)	0.02
		Geriatric rehabilitation unit	25 (34)	3 (16)	22 (40)	0.09
		Nursing home	16 (22)	3 (16)	13 (24)	0.75
Intended medical practice		In-hospital	7 (9)	5 (26)	2 (4)	0.01
		Private	25 (34)	5 (26)	20 (36)	0.77
		Mixed	22 (30)	7 (37)	15 (27)	0.43
		Unknown	20 (27)	2 (11)	18 (33)	0.08
Preference of specialty			57 (77)	14 (74)	43 (78)	0.75
In case of preference of specialty, which one?		General medicine	27 (36)	11 (58)	16 (29)	0.02
		Medical specialty	16 (22)	1 (5)	15 (27)	0.05
		Surgical specialty	6 (8)	0 (0)	6 (15)	0.32
		Gynecology/Pediatrics	6 (8)	0 (0)	6 (15)	0.32
		Emergency medicine	2 (3)	2 (11)	0 (0)	0.06
		Geriatrics	0 (0)	0 (0)	0 (0)	1
Choice of geriatric CIHT by conviction			52 (70)	17 (89)	35 (63)	0.04
First experience in geriatrics			31 (42)	11 (58)	20 (36)	0.10
Aware of the DSS in geriatrics			45 (61)	13 (68)	32 (58)	0.43
Students’ opinion on availability of information about DSS	Information on DSS considered sufficient		3 (4)	2 (11)	1 (2)	0.16
	Information on DSS considered not sufficient		71 (96)	17 (89)	54 (98)	0.16
	If not sufficient, wishes to receive information in the form of:	Website dedicated to the DSS	15 (20)	6 (35)	9 (16)	0.10
		Communication from the university	56 (76)	9 (52)	47 (85)	0.00
		Exchange with professionals and GPs	25 (34)	2 (12)	23 (42)	0.02
		Compulsory CIHT in geriatrics	2 (3)	1 (6)	1 (2)	0.42

N: number; GDSSG+: Diploma of Specialized Studies in Geriatrics intended; GDSSG−: Diploma of Specialized Studies in Geriatrics not intended; MM1: master’s degree in medicine 1st year; MM2: master’s degree in medicine 2nd year; MM3: master’s degree in medicine 3rd year; CIHT: clinical in-hospital training; DSS: Diploma of Specialized Studies; GP: General Practitioner. *p*: comparison of GDSSG+ to GDSSG−.

**Table 2:** Criteria for choosing a future specialty.

Parameter	Total (N = 74)	GDSSG+ (N = 19)	GDSSG− (N = 55)	*p*
	N (%)	N (%)	N (%)	
Varied activity	66 (89)	19 (100)	47 (85)	0.10
Intellectual attractiveness	54 (73)	14 (74)	40 (73)	1
Comprehensive patient care	47 (64)	15 (79)	32 (58)	0.17
Possible private medical practice	47 (64)	11 (58)	36 (65)	0.55
Possibility of carrying out additional training courses	41 (55)	12 (63)	29 (53)	0.43
Flexible working hours	39 (53)	8 (42)	31 (56)	0.28
Variety of exercise patterns	35 (47)	9 (47)	26 (47)	0.99
Work in multidisciplinary teams	28 (38)	7 (37)	21 (38)	0.92
Opportunity to pursue a career at hospital	18 (24)	3 (16)	15 (27)	0.37
Geographic location	23 (31)	4 (21)	19 (35)	0.39
Technicality of the specialty	16 (22)	3 (16)	13 (24)	0.75
Possibility to have an educational activity	15 (20)	3 (16)	12 (22)	0.67
Possibility of doing a post-intern formation *	16 (22)	0 (0)	16 (29)	0.01
High earnings	12 (16)	0 (0)	12 (22)	0.03
Possibility of partnerships with foreign countries	10 (14)	1 (5)	9 (16)	0.44
Possibility of doing research	8 (11)	0 (0)	8 (15)	0.10
Relational dimension	3 (4)	0 (0)	3 (5)	0.56

N: number; GDSSG+: specialized geriatric studies diploma group intended; GDSSG−: specialized geriatric studies diploma group not intended. *p*: comparison of GDSSG+ to GDSSG− * For example, physician assistant of the hospitals.

**Table 3:** Strengths of geriatrics according to students before and after CIHT for the GDSSG+ or GDSSG−.

	Before CIHT			*p* *	After CIHT			*p* #	*p* $
Parameter	GDSSG+ (N = 19)	GDSSG− (N = 55)	Total (N = 74)		GDSSG+ (N = 19)	GDSSG− (N = 55)	Total (N = 74)		
	N (%)	N (%)	N (%)		N (%)	N (%)	N (%)		
Intellectual Attractiveness	11 (58)	21 (38)	32 (43)	0.13	13 (68)	24 (44)	37 (50)	0.06	0.41
Technicality of the specialty	2 (11)	4 (7)	6 (8)	0.64	2 (11)	4 (7)	6 (8)	0.64	1
Varied activity	15 (79)	40 (73)	55 (74)	0.76	15 (79)	35 (63)	50 (68)	0.02	0.37
Variety of exercise patterns	10 (53)	27 (49)	37 (50)	0.79	11 (58)	30 (54)	41 (55)	0.80	0.51
Specialty of the future	10 (53)	29 (53)	39 (53)	0.99	11 (58)	32 (58)	43 (58)	0.98	0.51
Comprehensive patient care	18 (95)	50 (91)	68 (92)	1	18 (95)	51 (93)	69 (93)	1	1
Richness of ethical reflection	16 (84)	44 (80)	60 (81)	1	17 (89)	43 (78)	60 (81)	0.50	1
Opportunity of pursuing a career at hospital	2 (11)	14 (25)	16 (22)	0.21	3 (16)	12 (22)	15 (20)	0.75	0.84
Possibility of doing research	1 (5)	6 (11)	7 (9)	0.67	1 (5)	9 (16)	10 (14)	0.44	0.44
Possibility to have an educational activity	2 (11)	16 (29)	18 (24)	0.13	2 (11)	0 (0)	17 (23)	0.06	0.85
Flexible working hours	0 (0)	4 (7)	4 (5)	0.57	0 (0)	3 (5)	3 (4)	0.57	1
Multidisciplinary teamwork	15 (79)	39 (71)	54 (73)	0.56	16 (84)	44 (80)	60 (81)	1	0.24
Satisfactory salary	0 (0)	3 (5)	3 (4)	0.56	1 (5)	5 (9)	6 (8)	1	0.44
Opportunities for foreign partnerships	0 (0)	3(5)	3 (4)	0.56	0 (0)	3 (5)	0 (0)	0.57	0.25
Possibility to do a postintern formation	2 (11)	10 (18)	12 (16)	0.72	2 (11)	13 (24)	15 (20)	0.33	0.52
Possibility of carrying out additional training courses	2 (11)	10 (18)	12 (16)	0.72	4 (21)	12 (22)	16 (22)	1	0.40

CIHT: clinical in-hospital training; N: number; GDSSG+: specialized geriatric studies diploma group intended; GDSSG−: specialized geriatric studies diploma group not intended; *N*: number. *p* *: comparison of the GDSSG+ group to the GDSSG− group before the course *p* ^#:^ comparison of the GDSSG+ group to the GDSSG− group after the course. *p* ^$^: Comparison of total preplacement population group to total postplacement population group. For example, physician assistant of the hospitals.

**Table 4:** Negative points of geriatrics according to students before and after CIHT for the GDSSG+ or GDSSG−.

	Before CIHT			*p* *	After CIHT			*p* #	*p* $
Parameter	GDSSG+ (N = 19)	GDSSG− (N = 55)	Total (N = 74)		GDSSG+ (N = 19)	GDSSG− (N = 55)	Total (N = 74)		
	N (%)	N (%)	N (%)		N (%)	N (%)	N (%)		
Intellectually unattractive activity	0 (0)	3 (5)	3 (4)	0.57	0 (0)	3 (5)	3 (4)	0.57	1
Not technical enough	2 (11)	7 (13)	9 (12)	1	1 (5)	9 (16)	10 (14)	0.44	0.81
Too much variety	0 (0)	7 (13)	7 (9)	0.18	1 (5)	6 (11)	7 (9)	0.67	1
Not enough variety	0 (0)	3 (5)	3 (4)	0.57	0 (0)	3 (5)	3 (4)	0.57	1
Few opportunities for private medical practice	11 (58)	25 (45)	36 (49)	0.35	13 (68)	26 (47)	39 (53)	0.11	0.62
Too broad discipline	2 (11)	6 (11)	8 (11)	1	1 (5)	7 (13)	8 (11)	0.67	1
Too strong emphasis on the social dimension	1 (5)	12 (22)	13 (18)	0.16	0 (0)	14 (25)	14 (19)	0.02	0.83
Too much time for families	1 (5)	6 (11)	7 (9)	0.67	0 (0)	9 (16)	9 (12)	0.10	0.60
Dealing with old age/disability/MNCDs	8 (42)	33 (60)	41 (55)	0.18	8 (42)	35 (64)	43 (58)	0.10	0.74
Confrontation with death	7 (37)	27 (49)	34 (46)	0.36	6 (32)	26 (47)	32 (43)	0.23	0.74
Frustratingly limited diagnostic and therapeutic options	11 (58)	33 (60)	44 (59)	0.87	12 (63)	33 (60)	45 (61)	0.81	0.87
Little opportunity for research	0 (0)	3 (5)	3 (4)	0.57	0 (0)	4 (7)	4 (5)	0.57	1
Multidisciplinary teamwork	1 (5)	0 (0)	1 (1)	0.26	0 (0)	0 (0)	0 (0)	1	1
Insufficient earnings	0 (0)	1 (2)	1 (1)	1	0 (0)	2 (4)	2 (3)	1	1
Too few foreign partnerships	0 (0)	2 (4)	2 (3)	1	1 (5)	0 (0)	1 (1)	0.26	1
Poor knowledge of geriatrics	2 (11)	22 (40)	24 (32)	0.02	2 (11)	21 (38)	27 (36)	0.03	0.60
Working with the elderly perceived as low value	2 (11)	12 (22)	14 (19)	0.50	2 (11)	9 (16)	11 (15)	0.72	0.51
Lack of prestige of the specialty	2 (11)	10 (18)	12 (16)	0.72	1 (5)	13 (24)	14 (19)	0.10	0.67
Few opportunities for postintern formation	0 (0)	0 (0)	0 (0)	1	0 (0)	0 (0)	0 (0)	1	1
Few opportunities for further training	0 (0)	1 (2)	1 (1)	1	0 (0)	0 (0)	0 (0)	1	1
Lack of human and financial resources	0 (0)	0 (0)	0 (0)	1	0 (0)	1 (2)	1 (1)	1	1
Limitation to the elderly	0 (0)	0 (0)	0 (0)	1	0 (0)	1 (2)	1 (1)	1	1

CIHT: clinical in-hospital training; N: number; GDSSG+: specialized geriatric studies diploma group intended; GDSSG−: specialized geriatric studies diploma not intended. For example, physician assistant of the hospitals. *p* *: comparison of the GDSSG+ group to the GDSSG− group before the course. *p* ^#^: comparison of the GDSSG+ group to the GDSSG− group after the course. *p* ^$^: Comparison of total preplacement population group to total postplacement population group.

**Table 5:** Impact of in-hospital training on students’ view of geriatrics.

Parameter	Total (N = 74)	GDSSG+ (N = 19)	GDSSG− (N = 55)	*p*
	N (%)	N (%)	N (%)	
Positive evolution of the view of geriatrics	55 (74)	17 (89)	38 (69)	0.13
Positive change in the way the elderly are treated	70 (95)	19 (100)	51 (93)	0.57
Positive impact of the theoretical education provided on the view of geriatrics	60 (81)	18 (95)	42 (76)	0.10
Positive impact of the theoretical education provided on how to care for the elderly	63 (85)	18 (95)	45 (81)	0.27
Positive impact of clinical teaching on the view of geriatrics	59 (80)	18 (95)	41 (75)	0.10
Positive impact of clinical education on how to care for the elderly	67 (91)	18 (95)	49 (89)	0.67
Positive change in the view of geriatrics thanks to medical and health care team	54 (73)	16 (84)	38 (69)	0.25
Positive change in the way the elderly are treated with medical and health care team	65 (88)	19 (100)	46 (84)	0.10
Sufficient medical supervision	66 (89)	15 (79)	51 (93)	0.19
Pleasant welcome from the health care team	73 (99)	19 (100)	54 (98)	1
Sufficient impregnation of a geriatric culture	69 (93)	18 (95)	51 (93)	1
Recommendation of CIHT to other external students	69 (93)	17 (89)	52 (95)	0.60

GDSSG+: specialized geriatric studies diploma group intended; GDSSG−: specialized geriatric studies diploma group not intended; N: number; CIHT: clinical in-hospital training. *p*: comparison between GDSSG+ and GDSSG−.
